# Prediction and Optimization Algorithm for Intersection Point of Spatial Multi-Lines Based on Photogrammetry

**DOI:** 10.3390/s22249821

**Published:** 2022-12-14

**Authors:** Chengli Zhao, Hao Xiao, Zhangyan Zhao, Guoxian Wang

**Affiliations:** 1 School of Transportation and Logistics Engineering, Wuhan University of Technology, Wuhan 430063, China; 2 CCCC Second Harbor Engineering Company Ltd., Wuhan 430040, China; 3 Key Laboratory of Large-Span Bridge Construction Technology, Wuhan 430040, China; 4 Research and Development Center of Transport Industry of Intelligent Manufacturing Technologies of Transport Infrastructure, Wuhan 430040, China

**Keywords:** photogrammetry, intersection, information entropy, iterative optimization, straight line

## Abstract

The basic theory of photogrammetry is mature and widely used in engineering. The environment in engineering is very complex, resulting in the corners or multi-line intersections being blocked and unable to be measured directly. In order to solve this problem, a prediction and optimization algorithm for intersection point of spatial multi-lines based on photogrammetry is proposed. The coordinates of points on space lines are calculated by photogrammetry algorithm. Due to the influence of image point distortion and point selection error, many lines do not strictly intersect at one point. The equations of many space lines are used to fit their initial value of intersection point. The initial intersection point is projected onto each image, and the distances between the projection point and each line on the image plane are used to weight the calculated spatial lines in combination with the information entropy. Then the intersection point coordinates are re-fitted, and the intersection point is repeatedly projected and recalculate until the error is less than the threshold value or reached the set number of iterations. Three different scenarios are selected for experiments. The experimental results show that the proposed algorithm significantly improves the prediction accuracy of the intersection point.

## 1. Introduction

Cranes are important equipment for the development of modern industry and are widely used in ports, metallurgy, urban construction, aerospace, petrochemical and other fields. The role of the crane in the process of cargo transportation is irreplaceable as it can transport high-load cargo, such as the hoisting of offshore drilling platforms, the assembly of heavy ships, and the hoisting of nuclear power plant containment domes [1,2,3,4,5].

With the continuous development of hydraulic technology [6], computer technology [7], communication technology [8], advanced control technology [9], and new energy technology [10,11,12], the intelligence level of cranes is also constantly improving, but its structural safety [13] is always an important part of its safety. Once there is a problem with the structural safety of large construction machinery, it is easy to cause significant loss of life and property [14]. Therefore, large-scale construction machinery such as hoisting machinery is also a highly dangerous mechanical equipment [15].

In the past 20 years, safety assessment has been gradually applied in structural engineering, chemical disasters, information security, and other fields, while there have been relatively few safety assessment theories for cranes [16,17,18]. The load of the crane is usually large, ranging from several tons to thousands of tons, and the lifting height ranges from tens of meters to hundreds of meters [19]. Therefore, with the increasing application of cranes, the number of accidents is also rising. Therefore, it is very important to carry out safety assessment and risk monitoring of cranes.

The structural safety assessment of cranes refers to the assessment of the potential hazards and severity of mechanical structures. Commonly used crane risk assessment methods include fuzzy comprehensive assessment method, risk assessment method based on combination weighting, comprehensive risk assessment method, etc. [20]. But no matter which risk assessment method is used, key dimensions or the coordinates of key points need to be obtained. For construction machinery in complex working environments, truss is a common local structure. The key points on the crane usually show a face on the image, so the selection of measuring tools is particularly critical. Especially for the complex local structure of the crane, the design scheme of the engineering test is also critical, which is related to whether the key point coordinates can be accurately measured. After the key points are measured, they can be used as the basic data for crane safety evaluation. These data will play an important role in crane safety evaluation.

In the field of engineering, the commonly used measurement methods are: coordinate measuring machine, articulated arm measuring machine, laser tracker, structured light measurement system, total station, laser scanner, photogrammetry, etc. [21]. The three-coordinate measuring machine (CMM) has high measurement accuracy, good flexibility, and strong reverse engineering capabilities [22]. It is widely used in the mold industry, and it is a modern intelligent tool that integrates design, testing, and statistical analysis. As a portable measuring device, the articulated arm measuring machine needs to touch the point to be measured in space to complete the measurement [23]. The laser tracker uses a spherical coordinate system and relies on single-frequency laser interferometric ranging, which has high measurement accuracy and fast measurement speed. Moreover, laser tracker has certain advantages in large scenes [23]. Structured light measurement systems are divided into line structured light measurement systems and surface structured light measurement systems, which use the principle of triangulation to obtain the three-dimensional coordinates of points [24]. The total station is a high-precision measurement device that integrates light, machinery, and electricity. Its basic measurement principle is the same as that of the laser tracker, but the distance measurement method is different. Its measurement distance is long and its application range is very wide [25]. Laser scanners use a large number of laser points to form point clouds, so that three-dimensional information of the outer surface of the object to be measured can be obtained [26]. Photogrammetry is based on the relative positional relationship between the photographic center, image point, and spatial point, using two or more images to complete the coordinates calculation of the spatial points, and the measurement distance can be close or far [27]. Among the above measurement methods, the CMM, the articulated arm measurement machine, and the structured light measurement system have shorter measurement distances. Both the articulated arm measuring machine and the laser tracker are contact measuring methods. The operation of the total station is complicated, and the coordinates of a large number of points must be measured one by one. Laser scanners are extremely expensive to purchase and expensive to maintain. None of these measurement methods can meet the measurement needs of large construction machinery such as port machinery. The structure of large construction machinery such as port machinery is usually relatively tall, the range to be measured is relatively wide, and its working environment is complex. These factors determine that the measurement method that can be widely used in port machinery and other large construction machinery must be efficient, non-contact, portable, and have long measuring distance. Among the existing measurement methods, photogrammetry is undoubtedly the most advantageous. With the development of computer technology and artificial intelligence technology, the accuracy and stability of image-based visual measurement are also constantly improving. The intersection of close-range photogrammetry and advanced technologies such as computer vision is getting deeper and deeper, so the application range of close-range photogrammetry is also expanding [28].

The demand for close-up photogrammetry in aerospace [29], automobile manufacturing [30], mold manufacturing [31], material science [32], biomedicine [33], cultural heritage protection [34], etc. is increasing. After close-range photogrammetry entered the digital stage in 1980, many digital close-range photogrammetry systems appeared. There are TRITOP system of German GOM company, DPA-Pro system of German AICON 3D company, etc., whose technologies all come from the IAPG Institute. In addition, there are the V-STARS system of GSI Corporation of the United States, the Australis system of Photometrix Corporation of Australia, and the Metronor system of Metronor Corporation of Norway. The V-STARS system was developed by GSI Corporation of the United States and is currently the most mature commercial digital close-range industrial photogrammetry system in the world [35]. The Lensphoto multi-baseline digital close-up photogrammetry system, which was directly participated and launched by Academician Zhang Zuxun of Wuhan University, has been applied in the fields of water conservancy and electric power measurement, building measurement, cultural relics protection, and other fields [36].

## 2. Related Work

The application of close-range photogrammetry in port machinery and other large construction machinery is also increasing. Lu Enshun from Wuhan University of Technology [37] developed a photogrammetry-based radius measurement algorithm for the structural characteristics of port machinery, he used the approximate distortion model of the camera to determine the weight of each midperpendicular, and compared the equal-weight fitting algorithm with the weighted fitting algorithm. Wang Qi [38] developed a focal length calibration algorithm based on photogrammetry for port cranes. The central idea is to obtain image information through the Internet of Things technology, and then obtain the attitude parameters by minimizing the error function. Lin Xuanxiang [39] disclosed a method for detecting the deformation of the main beam of a hoisting machine based on photogrammetry, which uses photogrammetry to obtain the coordinates of the point to be measured, and then fits a curve according to the obtained coordinates to determine the deformation of the main beam. At present, the application of photogrammetry in large-scale construction machinery is relatively small, and there is a relatively broad research space.

In the theory of close-range photogrammetry [40], there are usually observation errors, so redundant observations are introduced to increase the accuracy of the calculation. Using multiple images to complete the space intersection is a commonly used adjustment method. Li Zhongmei added the overall least squares estimation in the process of multi-image space intersection and removed the gross error. Li Jiatian [41] combined the space intersection with the neural network to reduce the influence of nonlinear errors on solving three-dimensional coordinates. Faugeras and Mourrain [42] creatively gave a new derivation of the three-focal tensor equation, using three images to complete the solution; this method has been widely used in various fields. In the optimal solution triangulation of three views, Stewenius et al. used the method of interactive algebra to solve the polynomial matrix to obtain the triangulation result [43]. Agarwal et al. addressed the problem of global triangulation using fractional programming methods [44]. Dai et al. proposed a norm-based optimization method to improve the efficiency of triangulation [45]. In the multi-view triangulation, Zhang et al. selected some observation vectors as subsets, and then solved the subset data, which also improved the efficiency of triangulation [46].

Entropy is a thermodynamic concept proposed by physicist R. Clausius. C.E.Shannon first introduced entropy in thermodynamics into information theory. The appearance of information entropy is a sign of the generalization of entropy. It is widely used in physics, chemistry, medicine, water conservancy, communication, and mechanical safety assessment [47]. Since information entropy can measure the uncertainty of the appearance of things, it is also widely used in image processing to increase the accuracy of feature extraction, but there are few studies that can combine the basic principles of information entropy and photogrammetry.

For the safety assessment of large machinery such as port machinery, critical dimensions or the coordinates of key points are very important. Due to the limitation of the shooting angle or the occlusion of other mechanical structures, the key points are likely to be occluded or cannot be directly measured. Inspired by previous studies by scholars, the key points for dimension measurement or safety assessment are generally the intersection points of three straight lines or corner points with obvious features [48,49]. Combining structural features of large construction machinery, a prediction and optimization algorithm for the intersection point of multiple space straight lines is proposed [41,50,51]. In order to comprehensively consider the distortion of image points [52,53], the error of point selection, and the different shooting conditions of each image, the algorithm introduces information entropy [54,55]. Then the spatial lines involved in the calculation of fitting points are weighted [37] to further improve the fitting accuracy.

Therefore, compared with the previous work, the innovations of this paper are as follows: (1) we use multiple images to predict and optimize the intersection of straight lines; (2) an iterative optimization method based on reprojection is proposed; and (3) we introduce information entropy and weight the space line.

The rest of this paper is arranged as follows. Section 3.1 introduces the intersection point prediction algorithm of equal-weighted multiple lines. Section 3.2 introduces the intersection point prediction algorithm of weighted multiple lines. Section 4 introduces three experiments, all of which are used to verify the accuracy and stability of the algorithm in this paper. Section 5 provides a detailed and comprehensive analysis of the experimental results. Section 6 summarizes the entire paper.

## 3. Methodology

### 3.1. The Traditional Intersection Point Prediction Algorithm of Multiple Straight Lines

The manufacturing and installation process of the camera is very precise, but there will still be certain errors [56]. At the same time, when calculating the coordinates of the spatial points corresponding to the image points through the basic principle of photogrammetry [40], the selection of the image points will also have certain errors. Various factors lead to errors in the calculation of the spatial coordinates of the points to be measured. Hence, there will also be errors between the coordinates of the prediction intersection point and the ideal point.

**Figure 1:**
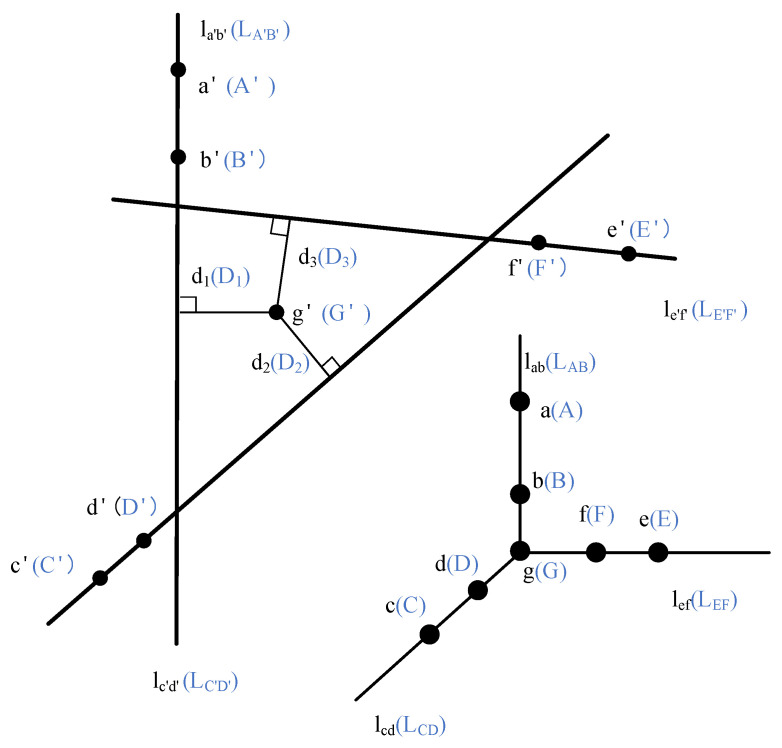
Schematic diagram of the intersection.

Taking the intersection point of three straight lines as an example, as shown in Figure 1, there are seven image points in the image plane, namely, *a*, *b*, *c*, *d*, *e*, *f*, and *g*, all of which are ideal image points, and the corresponding ideal space points are *A*, *B*, *C*, *D*, *E*, *F*, and *G*. The actual selected image points are $a^{\prime }$, $b^{\prime }$, $c^{\prime }$, $d^{\prime }$, $e^{\prime }$, $f^{\prime }$, and $g^{\prime }$, and the actual spatial points calculated by photogrammetry are $A^{\prime }$, $B^{\prime }$, $C^{\prime }$, $D^{\prime }$, $E^{\prime }$, $F^{\prime }$, and $G^{\prime }$. The straight line formed by points *a* and *b* is $l_{ab}$, the straight line formed by points *c* and *d* is $l_{cd}$, the straight line formed by points *e* and *f* is $l_{ef}$, and the straight lines $l_{ab}$, $l_{cd}$, and $l_{ef}$ intersect at point *g*. Due to optical distortion and point selection error, the straight line formed by points $a^{\prime }$ and $b^{\prime }$ is $l_{a^{\prime }b^{\prime }}$, the straight line formed by points $c^{\prime }$ and $d^{\prime }$ is $l_{c^{\prime }d^{\prime }}$, and the straight line formed by points $e^{\prime }$ and $f^{\prime }$ is $l_{e^{\prime }f^{\prime }}$. The straight lines $l_{a^{\prime }b^{\prime }}$, $l_{c^{\prime }d^{\prime }}$ and $l_{e^{\prime }f^{\prime }}$ will not strictly intersect at the point $g^{\prime }$. Whether it is the image plane where the image plane lines $l_{ab}$, $l_{cd}$, and $l_{ef}$ are located or the object space where the space straight lines $L_{AB}$, $L_{CD}$, and $L_{EF}$ are located, their schematic diagrams of the intersection point can both be represented by Figure 1. According to the basic principles of photogrammetry [40], the coordinates of the six spatial points $A^{\prime }$, $B^{\prime }$, $C^{\prime }$, $D^{\prime }$, $E^{\prime }$, and $F^{\prime }$ are obtained, and then the equations of the three straight lines $L_{A^{\prime }B^{\prime }}$, $L_{C^{\prime }D^{\prime }}$, and $L_{E^{\prime }F^{\prime }}$ are obtained according to the spatial coordinates of the six points. The three straight lines $L_{A^{\prime }B^{\prime }}$, $L_{C^{\prime }D^{\prime }}$, and $L_{E^{\prime }F^{\prime }}$ will not strictly intersect at the point $G^{\prime }$. To obtain the spatial coordinates of the fitting point $G^{\prime }$, a mathematical model is established to minimize the sum of the distances from the fitting point $G^{\prime }$ to the three spatial straight lines.

The distances from image point $g^{\prime }$ to image plane lines $l_{a^{\prime }b^{\prime }}$, $l_{c^{\prime }d^{\prime }}$, and $l_{e^{\prime }f^{\prime }}$ are $d_{1}$, $d_{2}$, and $d_{3}$, respectively. The distances from point $G^{\prime }$ to lines $L_{A^{\prime }B^{\prime }}$, $L_{C^{\prime }D^{\prime }}$, and $L_{E^{\prime }F^{\prime }}$ are $D_{1}$, $D_{2}$, and $D_{3}$, respectively. The solution formulas are as follows.

(1) $$\begin{array}{c} D_{1}=\{{\left[X_{G^{\prime }}-\left(X_{B^{\prime }}-X_{A^{\prime }}\right)m_{1}+X_{A^{\prime }}\right]}^{2}+{\left[Y_{G^{\prime }}-\left(Y_{B^{\prime }}-Y_{A^{\prime }}\right)m_{1}+Y_{A^{\prime }}\right]}^{2}\\ +{\left[Z_{G^{\prime }}-\left(Z_{B^{\prime }}-Z_{A^{\prime }}\right)m_{1}+Z_{A^{\prime }}\right]}^{2}{\}}^{1/2} \end{array}$$

(2) $$\begin{array}{c} D_{2}=\{{\left[X_{G^{\prime }}-\left(X_{D^{\prime }}-X_{C^{\prime }}\right)m_{2}+X_{C^{\prime }}\right]}^{2}+{\left[Y_{G^{\prime }}-\left(Y_{D^{\prime }}-Y_{C^{\prime }}\right)m_{2}+Y_{C^{\prime }}\right]}^{2}\\ +{\left[Z_{G^{\prime }}-\left(Z_{D^{\prime }}-Z_{C^{\prime }}\right)m_{2}+Z_{C^{\prime }}\right]}^{2}{\}}^{1/2} \end{array}$$

(3) $$\begin{array}{c} D_{3}=\{{\left[X_{G^{\prime }}-\left(X_{F^{\prime }}-X_{E^{\prime }}\right)m_{3}+X_{E^{\prime }}\right]}^{2}+{\left[Y_{G^{\prime }}-\left(Y_{F^{\prime }}-Y_{E^{\prime }}\right)m_{3}+Y_{E^{\prime }}\right]}^{2}\\ +{\left[Z_{G^{\prime }}-\left(Z_{F^{\prime }}-Z_{E^{\prime }}\right)m_{3}+Z_{E^{\prime }}\right]}^{2}{\}}^{1/2} \end{array}$$

where

(4) $$m_{1}=\frac{\begin{array}{c} \left(X_{B^{\prime }}-X_{A^{\prime }}\right)\left(X_{G^{\prime }}-X_{A^{\prime }}\right)+\left(Y_{B^{\prime }}-Y_{A^{\prime }}\right)\left(Y_{G^{\prime }}-Y_{A^{\prime }}\right)+\left(Z_{B^{\prime }}-Z_{A^{\prime }}\right)\left(Z_{G^{\prime }}-Z_{A^{\prime }}\right) \end{array}}{{\left(X_{B^{\prime }}-X_{A^{\prime }}\right)}^{2}+{\left(Y_{B^{\prime }}-Y_{A^{\prime }}\right)}^{2}+{\left(Z_{B^{\prime }}-Z_{A^{\prime }}\right)}^{2}}$$

(5) $$m_{2}=\frac{\begin{array}{c} \left(X_{D^{\prime }}-X_{C^{\prime }}\right)\left(X_{G^{\prime }}-X_{C^{\prime }}\right)+\left(Y_{D^{\prime }}-Y_{C^{\prime }}\right)\left(Y_{G^{\prime }}-Y_{C^{\prime }}\right)+\left(Z_{D^{\prime }}-Z_{C^{\prime }}\right)\left(Z_{G^{\prime }}-Z_{C^{\prime }}\right) \end{array}}{{\left(X_{D^{\prime }}-X_{C^{\prime }}\right)}^{2}+{\left(Y_{D^{\prime }}-Y_{C^{\prime }}\right)}^{2}+{\left(Z_{D^{\prime }}-Z_{C^{\prime }}\right)}^{2}}$$

(6) $$m_{3}=\frac{\begin{array}{c} \left(X_{F^{\prime }}-X_{E^{\prime }}\right)\left(X_{G^{\prime }}-X_{E^{\prime }}\right)+\left(Y_{F^{\prime }}-Y_{E^{\prime }}\right)\left(Y_{G^{\prime }}-Y_{E^{\prime }}\right)+\left(Z_{F^{\prime }}-Z_{E^{\prime }}\right)\left(Z_{G^{\prime }}-Z_{E^{\prime }}\right) \end{array}}{{\left(X_{F^{\prime }}-X_{E^{\prime }}\right)}^{2}+{\left(Y_{F^{\prime }}-Y_{E^{\prime }}\right)}^{2}+{\left(Z_{F^{\prime }}-Z_{E^{\prime }}\right)}^{2}}$$

$D_{1}$, $D_{2}$, and $D_{3}$ can be considered to be errors caused by camera distortion and point selection. Therefore, when the sum of $\left(D_{1}+D_{2}+D_{3}\right)$ reaches the minimum value, the coordinates of point $G^{\prime }$ are the desired coordinates. To facilitate the calculation, the minimum value of the sum of $\left(D_{1}+D_{2}+D_{3}\right)$ is converted into the minimum value of the sum of $\left({D_{1}}^{2}+{D_{2}}^{2}+{D_{3}}^{2}\right)$. The calculation formula is as follows.

(7) $$W\left(X_{G^{\prime }},Y_{G^{\prime }},Z_{G^{\prime }}\right)={D_{1}}^{2}+{D_{2}}^{2}+{D_{3}}^{2}$$

*W* is the function value, and the MATLAB optimization toolbox is used to constrain the nonlinear minimization function *W* to obtain coordinates of the point $G^{\prime }$. This method is the traditional algorithm (TA).

### 3.2. Intersection Point Prediction Algorithm of Weighted Multiple Lines

The initial coordinate value of the fitting point $G^{\prime }$ can be obtained from the formula in Section 3.1, and the corresponding image point $g^{\prime }$ can be obtained by projecting the spatial point $G^{\prime }$ onto the image. The calculation formula is as follows [40].

(8) $$x_{g^{\prime }}=-f\frac{\begin{array}{c} \left(\cos\varphi \cos\kappa -\sin\varphi \sin\omega \sin\kappa \right)\left(X_{g^{\prime }}-X_{S}\right)+\cos\omega \sin\kappa \left(Y_{g^{\prime }}-Y_{S}\right)\\ +\left(\sin\varphi \cos\kappa +\cos\varphi \sin\omega \sin\kappa \right)\left(Z_{g^{\prime }}-Z_{S}\right) \end{array}}{\begin{array}{c} -\sin\varphi \cos\omega \left(X_{g^{\prime }}-X_{S}\right)-\sin\omega \left(Y_{g^{\prime }}-Y_{S}\right)+\cos\varphi \cos\omega \left(Z_{g^{\prime }}-Z_{S}\right) \end{array}}-\Delta x$$

(9) $$y_{g^{\prime }}=-f\frac{\begin{array}{c} \left(-\cos\varphi \sin\kappa -\sin\varphi \sin\omega \sin\kappa \right)\left(X_{g^{\prime }}-X_{S}\right)+\cos\omega \cos\kappa \left(Y_{g^{\prime }}-Y_{S}\right)\\ +\left(\sin\varphi \cos\kappa +\cos\varphi \sin\omega \sin\kappa \right)\left(Z_{g^{\prime }}-Z_{S}\right) \end{array}}{\begin{array}{c} -\sin\varphi \cos\omega \left(X_{g^{\prime }}-X_{S}\right)-\sin\omega \left(Y_{g^{\prime }}-Y_{S}\right)+\cos\varphi \cos\omega \left(Z_{g^{\prime }}-Z_{S}\right) \end{array}}-\Delta y$$

where $X_{S}$, $Y_{S}$, $Z_{S}$, φ, ω, and κ are the exterior orientation elements of the camera, and Δx, Δy, and *f* are the intrinsic parameters of the camera.

When the error of the image point is larger, the distance from point $g^{\prime }$ to the straight line where the image point is located is also larger. When using multiple images to solve, due to the different shooting conditions of each image, the confidence of each image is different, and the confidence of the straight line composed of spatial points is also different. Thus, the information entropy is used to represent uncertainty. The larger the entropy value is, the smaller the weight of the indicator. The smaller the value is, the greater the weight. For *n* images, *m* space straight lines, the distance between the *j*th straight line on the *i*th image and the point $g^{\prime }$ on the image is denoted as $d_{g^{\prime }}\left(i,j\right)$, and there is a negative correlation between the weight $P_{e}$ of each spatial line and the corresponding $d_{g^{\prime }}$, that is, $P_{e}\propto {d_{g^{\prime }}}^{-1}$. Therefore, the information entropy formula of the *j*th straight line in space is as follows.

(10) $$H_{j}=-1/\ln n\sum_{j=1}^{n}(1/d_{g^{\prime }}\left(i,j\right))\ln\left(1/d_{g^{\prime }}\left(i,j\right)\right)$$

Then, the weight formula of the *j*th spatial straight line is as follows.

(11) $$P_{e}\left(j\right)=\frac{v_{j}}{\sum_{k=1}^{n}v_{k}}$$

where $v_{i}=1-H_{i}$.

Therefore, the formula for calculating the sum of the errors is as follows.

(12) $$W\left(X_{G^{\prime }},Y_{G^{\prime }},Z_{G^{\prime }}\right)=\sum_{j=1}^{m}(P_{e}\left(j\right){D_{j}}^{2})$$

where $D_{j}$ is the distance from point $G^{\prime }$ to the *j*th straight line.

**Figure 2:**
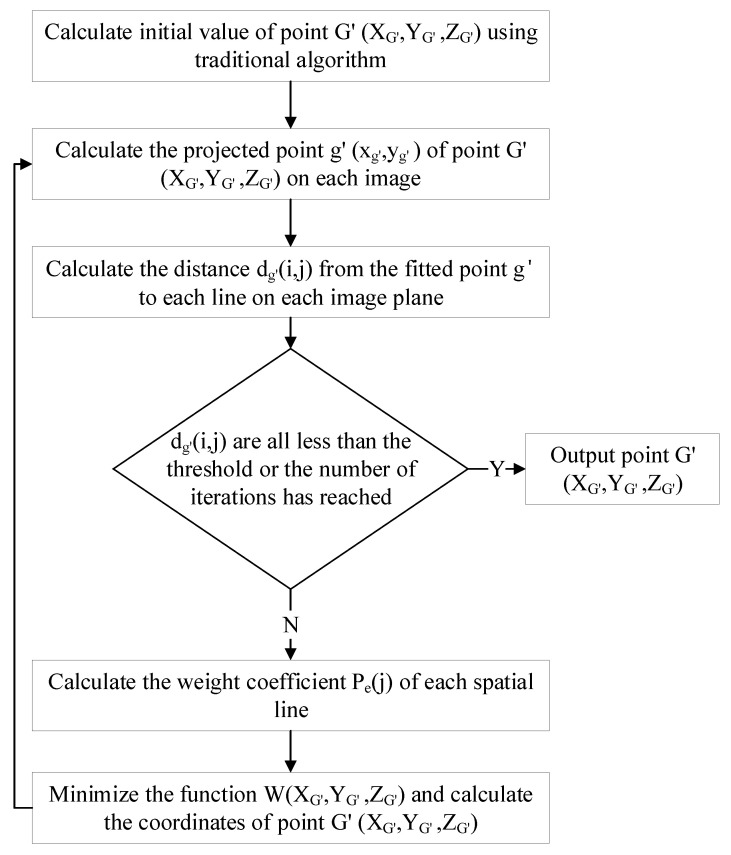
Iterative optimization calculation flow chart.

This method is called the weighted intersection point prediction and optimization algorithm (WIPPOA). The iterative calculation flow of this method is shown in Figure 2.

## 4. Case Study

### 4.1. Experimental Flow

**Figure 3:**
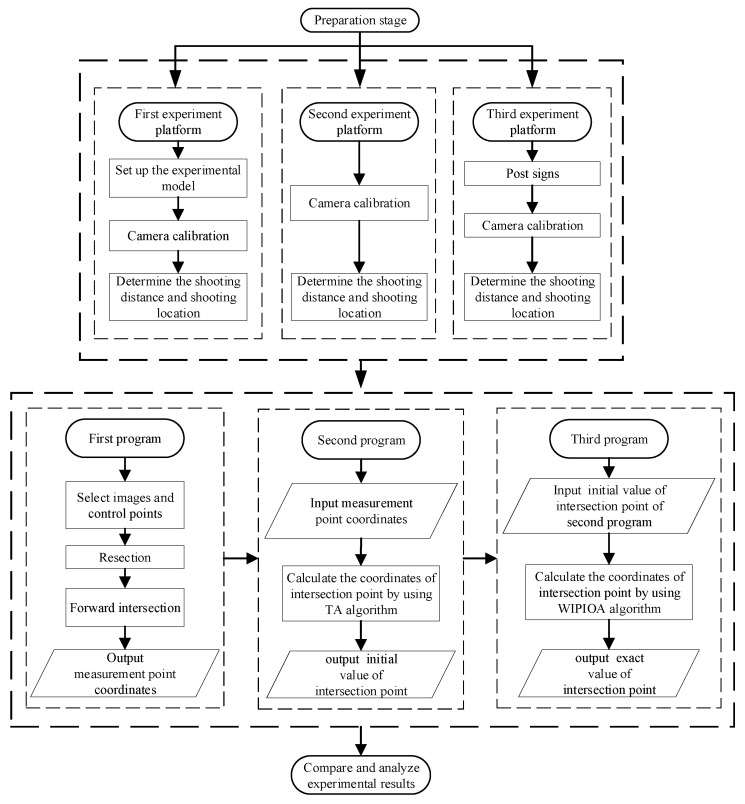
Experimental flow chart.

Since the effect of the algorithm can not be presented by computer simulation, the physical model is taken, and three images and three straight lines are taken as examples for verification experiments. In order to verify the stability and accuracy of the algorithm, three scenes are selected in the experiment. The first experimental scene is for the black and white square model, the feature points of the model are very clear. The second experiment is aimed at engineering scene, the object to be measured is shot without obvious identification points. The third experimental scene is also aimed at engineering scene, but this object to be measured is shot with clearly marked points. The experimental flow chart is in Figure 3. The coordinates of the points forming the lines are calculated based on the basic principles of photogrammetry [40], and then the equations of the three lines are calculated. Finally, the intersection point of the lines are fitted by the method of Section 3.1 and Section 3.2, and the experimental effects of the two algorithms are compared.

**Figure 4:**
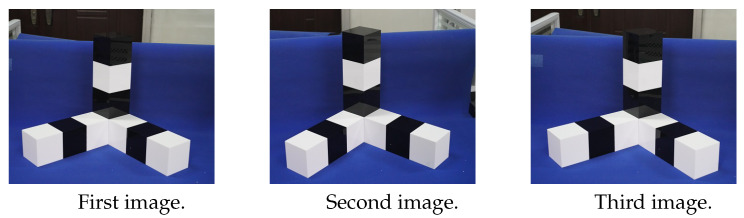
Images of the first experiment.

**Figure 5:**
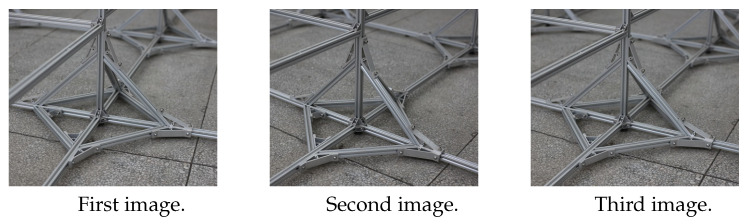
Images of the second experiment.

**Figure 6:**
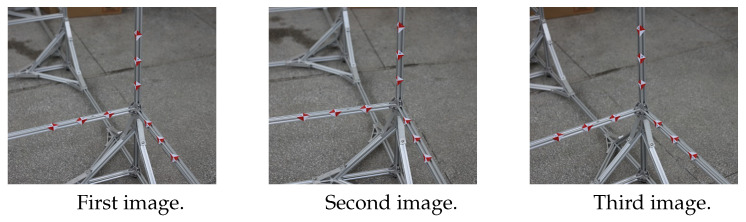
Images of the third experiment.

The three sets of photos taken in the experiment are shown in Figure 4, Figure 5 and Figure 6.

### 4.2. Experimental Platform

#### 4.2.1. First Experiment

**Figure 7:**
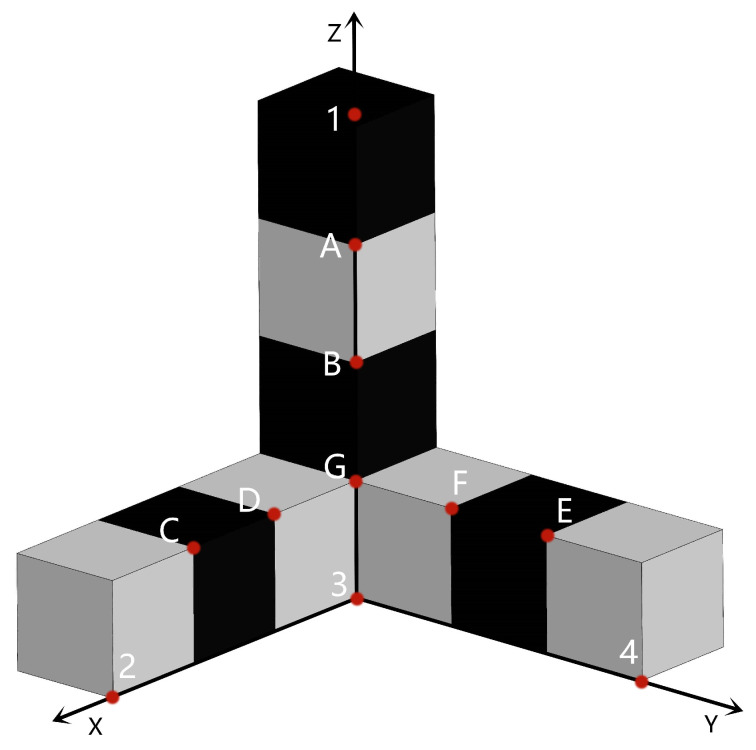
Points selection of first experiment.

As shown in Figure 7, the experimental model is composed of black and white squares with a side length of 100 mm. Points 1, 2, 3, and 4 are control points, and points *A*, *B*, *C*, *D*, *E*, F, and *G* are to be measured. Points *A* and *B* determine the straight line $L_{AB}$, points *C* and *D* determine the straight line $L_{CD}$, points *E* and *F* determine the straight line $L_{EF}$, and point *G* is the intersection point of the straight lines $L_{AB}$, $L_{CD}$, and $L_{EF}$. There will be a certain error between point $G^{\prime }$ fitted by the three straight lines and the ideal point *G*, and the error is used to measure the accuracy of the two methods.

The Sony A7R4A camera is used to complete the experiment, and the camera is calibrated by the MATLAB calibration tool. The camera parameters are shown in Table 1, where *f* is the focal length of the image, Δx and Δy are the principal point deviations of the image, $k_{1}$ and $k_{2}$ are the radial distortions, $q_{1}$ and $q_{2}$ are the tangential distortions, and $s_{1}$ and $s_{2}$ are the poise prism distortions.

The object space coordinates of the control points and points to be measured are shown in Table 2.

Three images are taken at three camera stations, as shown in Figure 4. According to the basic principle of photogrammetry [40], the three images are used to complete the calculation of the points to be measured.

The image plane coordinates of the three images are shown in Table 3.

The six exterior orientation elements of the three images are shown in Table 4.

The calculated coordinates of the space points are shown in Table 5.

#### 4.2.2. Second Experiment

**Figure 8:**
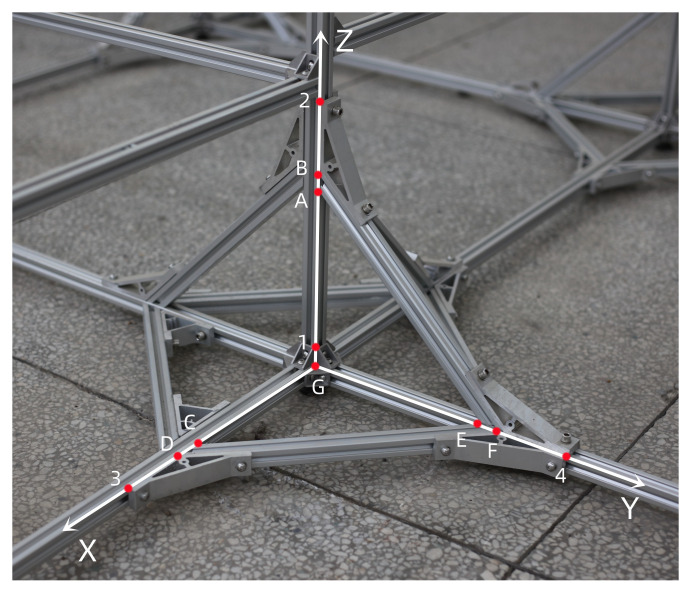
Points selection of second experiment.

As shown in Figure 8, points 1, 2, 3, and 4 are control points, and points *A*, *B*, *C*, *D*, *E*, *F*, and *G* are to be measured.

A Cannon 5DS camera is used to complete the experiment. The camera parameters are shown in Table 6. As shown in Figure 5, three images are taken at three camera stations.

The object space coordinates of the control points and points to be measured are shown in Table 7.

The image plane coordinates of the three images are shown in Table 8.

The six exterior orientation elements of the three images are shown in Table 9.

The calculated coordinates of the space points are shown in Table 10.

#### 4.2.3. Third Experiment

**Figure 9:**
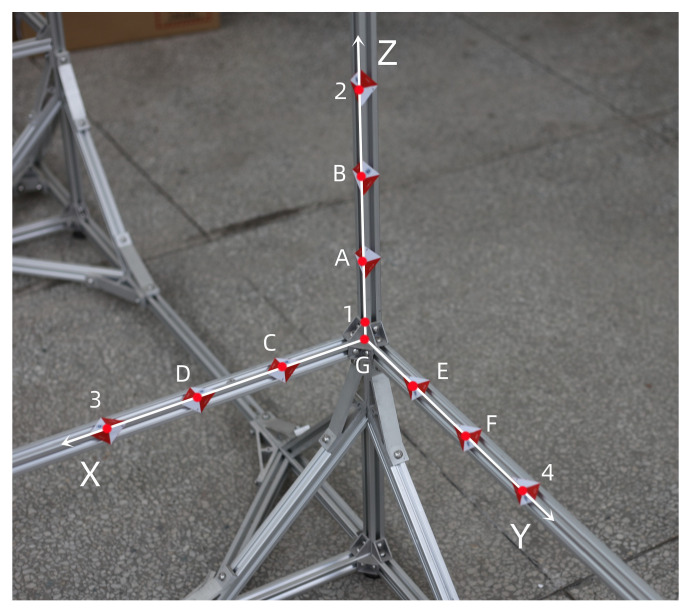
Points selection of third experiment.

As shown in Figure 9, points 1, 2, 3, and 4 are control points, and points *A*, *B*, *C*, *D*, *E*, *F*, and *G* are to be measured.

A Cannon 5DS camera is used to complete the experiment, and the camera parameters are shown in Table 11.

The object space coordinates of the control points and points to be measured are shown in Table 12.

The image plane coordinates of the three images are shown in Table 13.

The six exterior orientation elements of the three images are shown in Table 14.

The calculated coordinates of the space points are shown in Table 15.

## 5. Results and Discussion

The three experimental results of the weighted algorithm based on information entropy proposed in Section 3.2 are shown in Table 16, Table 17 and Table 18. Across 10 iterations, the object space coordinates of the fitting point $G^{\prime }$ and the weights of the space lines are updated.

Table 19 shows the coordinates of point $G^{\prime }$ obtained by algorithm TA and algorithm WIPPOA.

**Figure 10:**
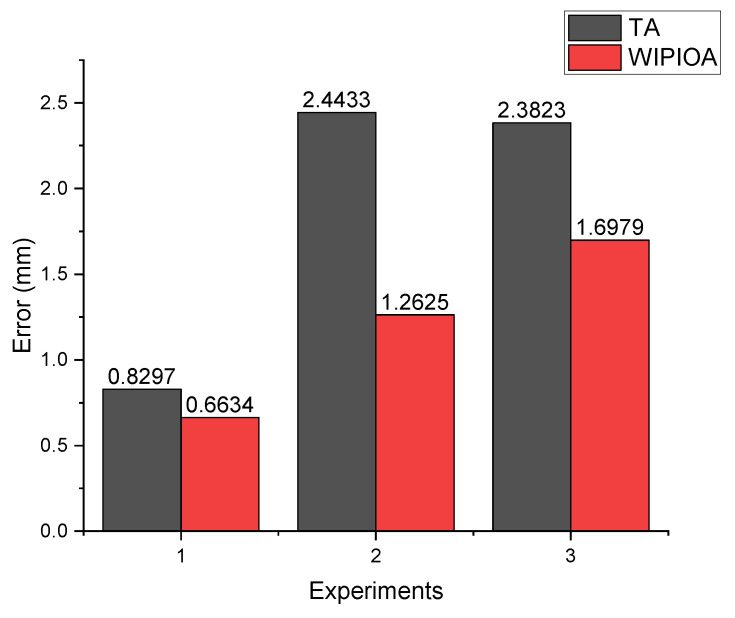
Error comparison.

The object of first experiment is a model with simple background and obvious feature points, so the error is small. The second experiment and the third experiment are engineering experiments with complex background, so the error is large. As can be seen from Table 16, Table 17 and Table 18, the algorithm can converge after a few iterations, and the convergence speed is relatively fast. As can be seen from Figure 10, the results of weighted iteration algorithm based on information entropy proposed in Section 3.2 are superior to equal weight iteration algorithm in all three scenarios. Therefore, the above experiments can show that the proposed algorithm can effectively improve the fitting accuracy of the intersection point, and has good stability and wide applicability.

## 6. Conclusions

Based on the basic theory of photogrammetry and the structural characteristics of large engineering machinery such as port machinery, a prediction and optimization algorithm for intersection point of spatial multi-Lines based on photogrammetry is proposed under the condition of considering the influence of many factors such as image point distortion and point selection error. The algorithm takes the spatial fitting point calculated by the traditional method as the initial point of weighted iteration. The projection points on each image are obtained from the spatial fitting points, and the distance between the projection points and each line in the image plane is combined with the information entropy to determine the weight of the space line, and then more accurate spatial coordinates of the intersection points can be obtained. Experimental results show that the proposed iterative optimization algorithm based on information entropy can significantly improve the accuracy of fitting points, and this algorithm has strong practicability.

Due to the limitations of experimental conditions and in order to better present comprehensive experimental data, the experiment in this paper only uses three images to calculate the intersection point. In subsequent studies, more images can be used to solve the problem to obtain more accurate fitting point coordinates. This method is not only applicable to port machinery, but also to other large engineering machinery. At the same time, this paper also introduces the information entropy in the proposed algorithm and uses information entropy to determine the relative weights. In future studies, we will focus on other structural characteristics of port machinery and other large machinery, and more a targeted algorithm will be put forward. We will make the photogrammetry applied more widely in engineering, at the same time, we will try to apply information entropy to more aspects of photogrammetry.

## Figures and Tables

**Table 1:** Camera parameters.

*f* (mm)	Resolution	Pixel Size (μm)	Δx (mm)	Δy (mm)	$k_{1}$	$k_{2}$
54.71	9504*63.362	3.7563	−0.0226	0.0984	−0.0671	2.4593

**Table 2:** The object space coordinates.

Points	X (mm)	Y (mm)	Z (mm)
1	0	0	400
2	300	0	0
3	0	0	0
4	0	300	0
A	0	0	300
B	0	0	200
C	200	0	100
D	100	0	100
E	0	200	100
F	0	100	100
G	0	0	100

**Table 3:** Image plane coordinates of the three images.

Points	First Image	Second Image	Third Image
1	(1.0354, −6.0228)	(−0.5533, −6.2137)	(0.8770, −14.1529)
2	(−5.9271, 11.1107)	(−9.8585, 9.8317)	(−8.4733, 2.3829)
3	(1.0595, 6.7681)	(−0.6178, 7.0176)	(0.5227, −0.5235)
4	(9.7984, 10.0437)	(6.1281, 11.2745)	(8.0912, 3.5829)
A	(1.0369, −2.6083)	(−0.5691, −2.7196)	(0.7723, −10.5186)
B	(1.0329, 0.6688)	(−0.6058, 0.6625)	(0.6766, −7.0399)
C	(−3.5038, 6.3139)	(−6.6579, 5.5119)	(−5.2699, −2.0842)
D	(−1.1359, 4.9890)	(−3.5317, 4.6911)	(−2.2423, −2.9200)
E	(6.7762, 5.7132)	(3.7242, 6.3093)	(5.4785, −1.3899)
F	(3.8126, 4.7242)	(1.4773, 5.0608)	(2.9457, −2.5926)

**Table 4:** Exterior orientation elements of three images.

Results	First Image	Second Image	Third Image
$X_{S}$ (mm)	1158.1825	848.6122	939.7586
$Y_{S}$ (mm)	901.3331	1207.0596	1110.4447
$Z_{S}$ (mm)	801.9554	713.2596	674.1236
φ (rad)	46.0246	2.0902	−0.9331
ω (rad)	0.6261	0.8783	2.3723
κ (rad)	−39.5504	4.2890	1.0692

**Table 5:** Result of space point coordinates.

Results	X (mm)	Y (mm)	Z (mm)
A	0.0923	−0.1268	300.1892
B	1.3620	0.9436	201.4292
C	200.9326	0.6234	101.7127
D	100.7434	0.0414	101.2665
E	1.7547	200.8025	101.8961
F	−0.2896	100.1147	100.7337

**Table 6:** Camera parameters.

*f* (mm)	Resolution	Pixel Size (μm)	Δx (mm)	Δy (mm)	$k_{1}$	$k_{2}$
54.58	8688*5792	4.1437	−0.0128	−0.0482	−0.1051	0.2600

**Table 7:** The object space coordinates.

Points	X (mm)	Y (mm)	Z (mm)
1	0	0	20
2	0	0	286
3	286	0	0
4	0	286	0
A	0	0	189
B	0	0	216
C	189	0	0
D	216	0	0
E	0	189	0
F	0	216	0
G	0	0	0

**Table 8:** Image plane coordinates of the three images.

Points	First Image	Second Image	Third Image
1	(0.2154, 1.3764)	(−0.9096, 0.9425)	(−1.9299, −5.5487)
2	(0.3845, −8.3994)	(−0.6524, −8.4335)	(−1.6810, −15.1306)
3	(−7.2398, 6.8973)	(−6.2938, 6.4930)	(−5.8707, 0.5994)
4	(10.1582, 5.6338)	(9.2210, 4.0027)	(8.7269, −3.1898)
A	(0.3609, −4.7909)	(−0.6998, −4.9935)	(−1.7236, −11.6007)
B	(0.3562, −5.7249)	(−0.7139, −5.9114)	(−1.7236, −12.5518)
C	(−4.5445, 5.1626)	(−4.3307, 4.7193)	(−4.4254, −1.4182)
D	(−5.2525, 5.5931)	(−4.8331, 5.1328)	(−4.8069, −0.9235)
E	(6.6608, 4.3270)	(5.7754, 3.1182)	(5.1525, −3.8039)
F	(7.6039, 4.6696)	(6.7049, 3.3669)	(6.1154, −3.6430)

**Table 9:** Exterior orientation elements of three images.

Results	First	Second	Third
$X_{S}$ (mm)	1028.3502	1214.2786	1252.0560
$Y_{S}$ (mm)	727.1096	574.6095	427.4130
$Z_{S}$ (mm)	680.8420	672.3328	696.8904
φ (rad)	11.5397	14.6075	−13.5447
ω (rad)	2.5925	0.3833	2.8993
κ (rad)	1.2498	−8.0723	32.8013

**Table 10:** Result of space points coordinates.

Results	X (mm)	Y (mm)	Z (mm)
A	1.7017	1.7258	193.5912
B	3.0446	1.8387	218.8006
C	189.3388	−2.8676	−2.0063
D	215.2834	−2.4519	−0.7051
E	2.2671	194.3242	3.7206
F	1.6158	219.9360	2.9717

**Table 11:** Camera parameters.

*f* (mm)	Resolution	Pixel Size (μm)	Δx (mm)	Δy (mm)	$k_{1}$	$k_{2}$
59.64	8688*5792	4.1437	−0.0067	−0.0363	−0.0910	−0.2109

**Table 12:** The object space coordinates.

Points	X (mm)	Y (mm)	Z (mm)
1	0	0	20
2	0	0	300
3	300	0	0
4	0	300	0
A	0	0	100
B	0	0	200
C	100	0	0
D	200	0	0
E	0	100	0
F	0	200	0
G	0	0	0

**Table 13:** Image plane coordinates of the three images.

Points	First Image	Second Image	Third Image
1	(−2.7266, −1.0344)	(1.1911, −0.2700)	(1.0960, −4.8962
2	(−2.9537, −9.2997)	(1.3194, −8.4048)	(1.1291, −13.0625)
3	(−11.7818, 2.6239)	(−8.4538, 1.6320)	(−8.3276, −2.2037)
4	(2.7505, 4.7265)	(4.0894, 6.3217)	(5.3035, 1.4577)
A	(−2.7947, −3.1986)	(1.2056, −2.4122)	(1.0894, −7.0475)
B	(−2.8676, −6.1531)	(1.2587, −5.3099)	(1.0960, −9.9291)
C	(−5.6159, 0.4847)	(−1.9918, 0.7257)	(−1.9702, −3.6781)
D	(−8.5921, 1.5352)	(−5.1826, 1.1816)	(−5.0989, −2.9534)
E	(−1.0727, 1.1045)	(2.0142, 2.0897)	(2.3123, −2.6232)
F	(0.7439, 2.8708)	(2.9922, 4.1148)	(3.7022, −0.6773)

**Table 14:** Exterior orientation elements of three images.

Results	First Image	Second Image	Third Image
$X_{S}$ (mm)	745.0881	425.2814	606.0294
$Y_{S}$ (mm)	1333.7530	1475.0074	1396.6841
$Z_{S}$ (mm)	1040.1013	1057.3479	1079.3126
φ (rad)	−44.6395	65.6167	−28.7047
ω (rad)	8.6576	0.9252	0.7950
κ (rad)	38.5326	−59.2604	28.8441

**Table 15:** Result of space points coordinates.

Results	X (mm)	Y (mm)	Z (mm)
A	−0.1471	0.2365	98.7990
B	0.8819	1.7787	199.8051
C	100.3537	−0.4227	0.1450
D	201.2386	2.6954	2.3575
E	0.3740	98.9673	0.4328
F	−0.0762	197.7977	−1.2015

**Table 16:** Results of first experiment.

Iteration	1	...	9	10
$P_{e}\left(1\right)$	0.27997144	...	0.27997711	0.27997711
$P_{e}\left(2\right)$	0.36192177	...	0.36191784	0.36191784
$P_{e}\left(3\right)$	0.35810679	...	0.35810505	0.35810505
$X_{G^{\prime }}$ (mm)	0.18107110	...	−0.12525462	−0.12525462
$Y_{G^{\prime }}$ (mm)	0.77749965	...	0.61155227	0.611552273
$Z_{G^{\prime }}$ (mm)	100.22591947	...	100.22466629	100.2246663

**Table 17:** Results of second experiment.

Iteration	1	...	9	10
$P_{e}\left(1\right)$	0.31479429	...	0.31524867	0.31524867
$P_{e}\left(2\right)$	0.31036264	...	0.30944042	0.30944042
$P_{e}\left(3\right)$	0.37484307	...	0.37531091	0.37531091
$X_{G^{\prime }}$ (mm)	−0.39151546	...	−0.88701586	−0.88701526
$Y_{G^{\prime }}$ (mm)	−2.27309467	...	−0.88701580	−0.88701520
$Z_{G^{\prime }}$ (mm)	−0.80592502	...	0.14265211	0.14265036

**Table 18:** Results of third experiment.

Iteration	1	...	9	10
$P_{e}\left(1\right)$	0.32706779	...	0.32701057	0.32701057
$P_{e}\left(2\right)$	0.32135332	...	0.32143443	0.32143443
$P_{e}\left(3\right)$	0.35157890	...	0.35155500	0.35155500
$X_{G^{\prime }}$ (mm)	−0.11845095	...	−1.19857656	−1.19857641
$Y_{G^{\prime }}$ (mm)	−2.37925704	...	−1.19857649	−1.19857635
$Z_{G^{\prime }}$ (mm)	0.02247442	...	0.09803921	0.09803913

**Table 19:** Coordinates update.

Point $G^{\prime }$	TA	WIPPOA
First experiment	(0.1811, 0.7775, 0.2259)	(−0.1253, 0.6116, 0.2247)
Second experiment	(−0.3915, −2.2731, −0.8059)	(−0.8870, −0.8870, 0.1427)
Third experiment	(−0.1185, −2.3793, 0.0225)	(−1.1986, −1.1985, 0.0980)

## Data Availability

Not applicable.
